# Thiopurine S-Methyltransferase and Methylenetetrahydrofolate Reductase Polymorphisms in Leukemia

**DOI:** 10.4274/tjh.2015.0001

**Published:** 2015-05-08

**Authors:** Serhan KÜPELİ

**Affiliations:** 1 Çukurova University Faculty of Medicine, Department of Pediatric Oncology and Pediatric Bone Marrow Transplantation Unit, Adana, Turkey

**Keywords:** Thiopurine S-methyltransferase, Methylenetetrahydrofolate reductase, Gene polymorphisms, Leukemia, childhood

## TO THE EDITOR

I read the paper by Belen et al. published in a recent issue of this journal with great interest [1]. They reported a 15-year-old girl with T-ALL who developed severe pancytopenia during consolidation and maintenance therapy despite dose reduction of 6-mercaptopurine (MP). They found thiopurine S-methyltransferase (TPMT) *3A/*3C polymorphisms upon TPMT genotyping. Prednisolone therapy produced a remarkable but transient bone marrow recovery in this patient.

The authors should be congratulated in that they followed the recent evidence-based clinical guidance recommendations related to safe and effective thiopurine administration. The authors began treatment with standard doses and down-titrated the MP according to the results of blood counts, and they omitted some parts of Protocol Ib and the second part of the reinduction therapy. Although in a recent systematic review 30%-50% of the normal dose for thiopurines in the case of heterozygous TPMT status was recommended [2], I agree with the authors about low-dose MP administration (2.5%-10% of normal dose) when necessary, because of the possibility of the presence of undetected deficiencies in other enzymes involved in the metabolic pathway [3]. Moreover, by omitting 6-thioguanine (6TG) treatment in reinduction therapy, the authors prevented the probable additional toxicity arising from another thiopurine drug since patients with heterozygous TPMT status have higher cytosolic 6TG nucleotide levels, the cytotoxic metabolites of both MP and 6TG [4].

On the other hand, after careful reading, some concerns and questions arose regarding the paper. First of all, it is not possible to understand that the patient had an accompanying methylenetetrahydrofolate reductase (MTHFR) polymorphism from either the title or the English abstract. Only after reading the Turkish abstract and the text I did realize that the patient had concomitant TPMT *3A/*3C and MTHFR C677T and A1298C polymorphisms, which were reported to be associated with increased myelotoxicity in children with ALL [5]. Although the authors stated that they found t(11;14) in karyotyping and that PCR tests for t(4;11) and t(9;21) were negative, they did not mention t(9;22), an important prognostic abnormality in patients with ALL. Additionally, the information about the extension of the disease at the time of diagnosis, such as involvement of the central nervous system (CNS) or kidneys and renal impairment due to increased tumor burden, is lacking in the case presentation. The latter two, if present, might be responsible for the protracted bone marrow suppression by increasing toxic effects of methotrexate. Furthermore, it is not clear to which risk category the patient was assigned in the treatment protocol in the text or from the figure (proper reading of the figure is impossible). I would want to learn more details regarding the clinical condition of the patient, such as whether she was given CNS radiotherapy or not, her renal function test results and especially those before methotrexate administration, and whether she completed the entire chemotherapy protocol or not. Lastly, I am curious about the follow-up results of this patient, since TPMT heterozygosity was reported to be associated with better event-free survival than in TPMT wild-type patients and thiopurine-induced cytopenias did not negatively affect the treatment outcome [6].
