# Supplementary material for: Thiopurine S-Methyltransferase and Methylenetetrahydrofolate Reductase Polymorphisms in Leukemia
Source: Turk J Haematol. 2015 May 8;32(2):184–5. doi: 10.4274/tjh.2015.0001 (PMC4451490; doi:10.4274/tjh.2015.0001)
Supplement: Related texts

# Reply: Authors' Response

**Authors:** Burcu Belen, Türkiz Gürsel

## REPYL

We would like to thank for comments on our article, entitled “Severe myelotoxicity associated with thiopurine S-methyltransferase *3A/*3C polymorphisms in a patient with pediatric leukemia and the effect of steroid therapy”, published in Turkish Journal of Hematology 2014, volume 31, issue 4, pages 276-285. 

Severe bone marrow suppression in our case occurred during consolidation chemotherapy which contained oral 6-mercaptopurine (6-MP), intravenous cyclophosphamide and low dose cytosine arabinoside and continued until the end of the maintenance treatment despite markedly reduced 6MP dose. It can not be attributed to renal impairment, central nervous system leukemia or methotrexate (MTX) toxicity because none of these were present during or before pancytopenia was noted. The patient is compound heterozygous for TPMT*3A and *3C polymorphisms, which is known to cause low enzyme activity and increase of intracellular thioguanine nucleotides, and lead to severe bone marrow suppression. Although Karas-Kuzelicki et al. reported that co-inheritance of MTHFR C677T with non-functional TPMT variants augments myelotoxicity during maintenance therapy with oral MTX and 6-MP in children with ALL [1], other investigators did not find significant association between these polymorphic variants and excessive myelotoxicity [2]. In line with these data, we did not observe severe leukopenia or hepatoxicity during the four courses of intensification therapy with high dose(5 g/m2) MTX, which was given in conjunction with adjusted dose 6-MP. This may be due to the presence of MTHFR A1298C polymorphism, which is associated with lower toxicity risk in patients with ALL. It has been suggested that linkage disequilibrium between C677T and A1298C may explain decreased toxicity during high dose MTX administration [3]. Possible effects of MTHFR C677T and A1298C polymorphisms on the stability of the TPMT enzyme were addressed in our article.

The patient was assigned to the medium risk group based on ALL-BFM-95 risk stratification criteria [4]. Renal function tests and glomerular filtration rate were within normal limits during all stages of chemotherapy. In regard to the cranial radiotherapy, 12 Gy prophylactic cranial irradiation was given before maintenance therapy without causing excessive myelosuppression. The patient is in complete remission for 3 years after completion of the maintenance chemotherapy.

Adding “MTHFR C677T and A1298C polymorphisms” to the title of our article was not possible due to the the word count restriction of Turkish Journal of Hematology.

Please kindly be informed that ‘t(9;21)’ expression in our article in Turkish Journal of Hematology 2014, volume 31, issue 4, pages 276-285 is incorrect. The correct expression should be t(9;22). We apologize for this typing error.

## References

[1] Karas-Kuzelicki N, Jazbec J, Milek M, Mlinaric-Rascan I (2009). Heterozygosity at the TPMT gene locus, augmented by mutated MTHFR gene, predisposes to 6-MP related toxicities in childhood ALL patients. Leukemia.

[2] Wall AM, Rubnitz JE (2003). Pharmacogenomic effects on therapy for acute lymphoblastic leukemia in children.. Pharmacogenomics J.

[3] Ongaro A, De Mattei M, Della Porta MG, Rigolin G, Ambrosio C, Di Raimondo F, Pellati A, Masieri FF, Caruso A, Catozzi L, Gemmati D (2009). Gene polymorphisms in folate metabolizing enzymes in adult acute lymphoblastic leukemia: effects on methotrexate-related toxicity and survival. Haematologica.

[4] Kocak U, Rosthøj S, Gursel T, Kaya Z, Aral YZ, Albayrak M, Keskin EY, Belen B, Isik M, Oner N (2012). ALL-BFM 95 treatment in Turkish children with acute lymphoblastic leukemia--experience of a single center. Pediatr Hematol Oncol.
